# Supplementary material for: A UMLS-based spell checker for natural language processing in vaccine safety
Source: BMC Med Inform Decis Mak. 2007 Feb 12;7:3. doi: 10.1186/1472-6947-7-3 (PMC1805499; doi:10.1186/1472-6947-7-3)
Supplement: Additional file 13 — RAP application directory. Sets up directory for RAP (RDF application for PHP) source code [file 1472-6947-7-3-S13.gz › rap/doc/phpdoc/__filesource/fsource_dataset__syntaxTriXSerializer.php.html]

xml version="1.0" encoding="iso-8859-1"?


File Source for TriXSerializer.php


# Source for file TriXSerializer.php

Documentation is available at TriXSerializer.php

```
1. <?php
2. // ----------------------------------------------------------------------------------
3. // Class: TriXSerializer
4. // ----------------------------------------------------------------------------------


8. /**
9. * Temporary implementation of a TriX-Serializer
10. *
11. *
12. * <BR><BR>History:<UL>
13. * <LI>06-02-2005                : First version of this class.</LI>
14. *
15. * @version  V0.9
16. * @author Daniel Westphal (http://www.d-westphal.de)
17. *
18. * @package     dataset
19. * @access    public
20. ***/
21. class TriXSerializer
22. {

24. /**
25. * Reference to the graphSet
26. *
27. * @var        object GraphSet
28. * @access    private
29. */
30. var $graphSet;


33. /**
34. * Constructor
35. * Needs a reference to a graphSet
36. *
37. * @param    GraphSet
38. * @access    public
39. */
40. function TriXSerializer(&$graphSet)
41. {
42. $this->graphSet=&$graphSet;
43. }

45. /**
46. * Serialize the dataset to a TriX string
47. *
48. * @return   string
49. * @access    public
50. */
51. function & serializeToString()
52. {
53. return $this->_serialize();
54. }

56. /**
57. * Serialize the dataset to a TriX string and save in file
58. *
59. * @param   string
60. * @access    public
61. */
62. function serializeToFile($fileName)
63. {
64. $serializedString=&$this->_serialize();
65. $handle = fopen($fileName, 'w');
66. fwrite($handle, $serializedString);
67. fclose($handle);
68. }


71. /**
72. * Serialize the dataset to a TriX string
73. *
74. * @return   string
75. * @access    private
76. */
77. function & _serialize()
78. {
79. //Trix header
80. $serializedString=
81. '<?xml version="1.0" encoding="utf-8"?>'.
82. '<TriX xmlns="http://www.w3.org/2004/03/trix/trix-1/">';

84. //serialize defaultGraph if it is not empty
85. $defaultGraph=& $this->graphSet->getDefaultGraph();
86. if ($defaultGraph->isEmpty()===false)
87. {
88. $serializedString.='<graph>';
89. for($iterator = $this->graphSet->findInDefaultGraph(null,null,null); $iterator->valid(); $iterator->next())
90. {
91. $serializedString.='<triple>';

93. $statement=$iterator->current();

95. $serializedString.=$this->_node2string($statement->getSubject());
96. $serializedString.=$this->_node2string($statement->getPredicate());
97. $serializedString.=$this->_node2string($statement->getObject());

99. $serializedString.='</triple>';
100. };
101. $serializedString.='</graph>';
102. }

104. //serialize namedGraphs
105. foreach ($this->graphSet->listGraphNames() as $graphName)
106. {
107. $serializedString.='<graph>';
108. $serializedString.='<uri>'.$graphName.'</uri>';
109. for($iterator = $this->graphSet->findInNamedGraphs(new Resource($graphName),null,null,null); $iterator->valid(); $iterator->next())
110. {
111. $serializedString.='<triple>';

113. $statement=$iterator->current();

115. $serializedString.=$this->_node2string($statement->getSubject());
116. $serializedString.=$this->_node2string($statement->getPredicate());
117. $serializedString.=$this->_node2string($statement->getObject());

119. $serializedString.='</triple>';
120. };
121. $serializedString.='</graph>';
122. };
123. //TriX footer
124. $serializedString.='</TriX>';
125. return $serializedString;
126. }

128. /**
129. * Serialize node to a TriX string
130. *
131. * @param Node
132. * @return   string
133. * @access    private
134. */
135. function _node2string($node)
136. {
137. switch ($node)
138. {
139. case (is_a($node,'BlankNode')):
140. return ('<id>'.$node->getLabel().'</id>');

142. case (is_a($node,'Resource')):
143. return ('<uri>'.$node->getLabel().'</uri>');

145. case (is_a($node,'Literal')):

147. if ($node->dtype!=null)
148. return ('<typedLiteral datatype="'.htmlentities($node->dtype).'">'.$node->getLabel().'</typedLiteral>');

150. if ($node->lang!=null)
151. return ('<plainLiteral xml:lang="'.htmlentities($node->lang).'">'.$node->getLabel().'</plainLiteral>');

153. return ('<plainLiteral>'.htmlentities($node->getLabel()).'</plainLiteral>');
154. }
155. }
156. }
157. ?>
```

Documentation generated on Thu, 7 Jul 2005 13:44:01 +0200 by phpDocumentor 1.3.0RC3
